# The complete chloroplast genome sequences of the *Rosa kokanica* (Regel) Regel ex Juz. (Rosaceae)

**DOI:** 10.1080/23802359.2021.1944363

**Published:** 2021-06-28

**Authors:** Tae-Young Choi, Aleksey Kim, Dong-Kap Kim, Su-Young Jung, Soo-Rang Lee

**Affiliations:** a Department of Biology Education, Chosun University, Gwangju, Republic of Korea; b Forest Biodiversity Division, Korea National Arboretum, Pocheon, Republic of Korea

**Keywords:** *Rosa kokanica*, complete chloroplast genome, Rosaceae

## Abstract

*Rosa kokanica* is a deciduous shrub distributed in Central Asia. We determined the genomic characteristics of the complete chloroplast genome in *R. kokanica* with a de novo assembly strategy. The chloroplast genome was 156,802bp in length harboring 89 protein coding genes, 37 tRNA genes and eight rRNA genes. It exhibits typical quadripartite structure comprising a large single-copy (LSC) (85,899bp), a small single-copy (SSC) (18,773bp) and a pair of inverted repeats (IRs) (26,065bp). Phylogenetic analysis of 16 chloroplast genomes from Rosaceae revealed that the genus *Rosa* is a monophyletic group and the *R. kokanica* is clustered together with the congener, *R. acicular.*

*Rosa kokanica* (Regel) Regel ex Juz. (Rosaceae) is a deciduous shrub distributed in high mountain slopes of Central Asia including Afghanistan, China (Xinjiang), Iran, Kazakhstan, Kyrgyzstan, Pakistan, Tadzhikistan and Uzbekistan (Gu and Robertson 2003). Genus *Rosa* is well known for its economic importance as ornamental and medicinal plants (Raymond et al. 2018). *R. kokanica,* which is one of ca. 30 *Rosa* taxa distributed in Central Asia, has been widely used for medicines, and has potential ornamental values (Tolekova et al. 2020). However, the genomic information applicable for Central Asian *Rosa* is still scarce. In the present study, we investigated the genomic architecture in the whole chloroplast genome of *R. kokanica* using whole genome shotgun sequencing.

We collected young leaves of *R. kokanica* from Namangang province, Uzbekistan (N 41°01′43.4″, E 70°36′27.5″). The voucher specimen was prepared and deposited at the Herbarium of Korea National Arboretum (KH) with the accession number KHB1547488. The total genomic DNA was extracted followed by manufacturer’s protocol (Qiagen, Hilden, Germany). After library preparation, the prepared libraries were sequenced on Illumina MiSeq platform (Illumina, San Diego, CA). Seven million high-quality 300 bp paired-end reads were obtained. We assembled 2.19GB reads with *de novo* strategy using Geneious Prime (ver. 4.2.1) according to the manufacturer’s instruction. The genes were predicted with GeSeq (Tillich et al. 2017), and manually curated based on Blast search result. The simple sequence repeats were investigated with MISA (Beier et al. 2017).

The complete chloroplast genome of *R. kokanica* has been submitted to Genbank (accession no. MW298478). It is 156,802bp in length with the typical quadripartite structure comprising a large single copy (LSC) (85,899bp), a small single-copy (SSC) (18,773bp) and a pair of inverted repeats (IRs) (26,065bp). The chloroplast genome contained 131 genes including 89 protein coding genes, 37 tRNA genes and eight rRNA genes. 51 simple sequence repeats were identified in the cp genome, most of which was mono-nucleotide repeat.

To investigate its phylogenetic relationship, the concatenated CDs sequences from entire chloroplast genome of 16 *Rosa* and outgroup taxa were aligned in MAFT (Katoh et al. 2019). All sequences except *R. kokanica* were downloaded from NCBI Genbank. We assigned *Dasiphora* and *Fragaria* as an outgroup following phylogenetic relationships based on a previous study (Potter et al. 2007). We inferred the phylogeny using Maximum likelihood (ML) algorithm implemented in RAxML v. 4.0 with GTR GAMMA model. For the clade support, 1000 bootstrap replicates were used. The 13 species of *Rosa* formed a monophyletic group (BP = 100) with strong support on ML tree (Figure 1). In ML tree, section *Pimpinellifoliae* did not form a monophyletic group, which is consistent with previous phylogenetic studies (Fougère-Danezan et al. 2015). The ML tree also indicated that *R. kokanica* is most closely related with *R. acicularis*.

**Figure 1.**
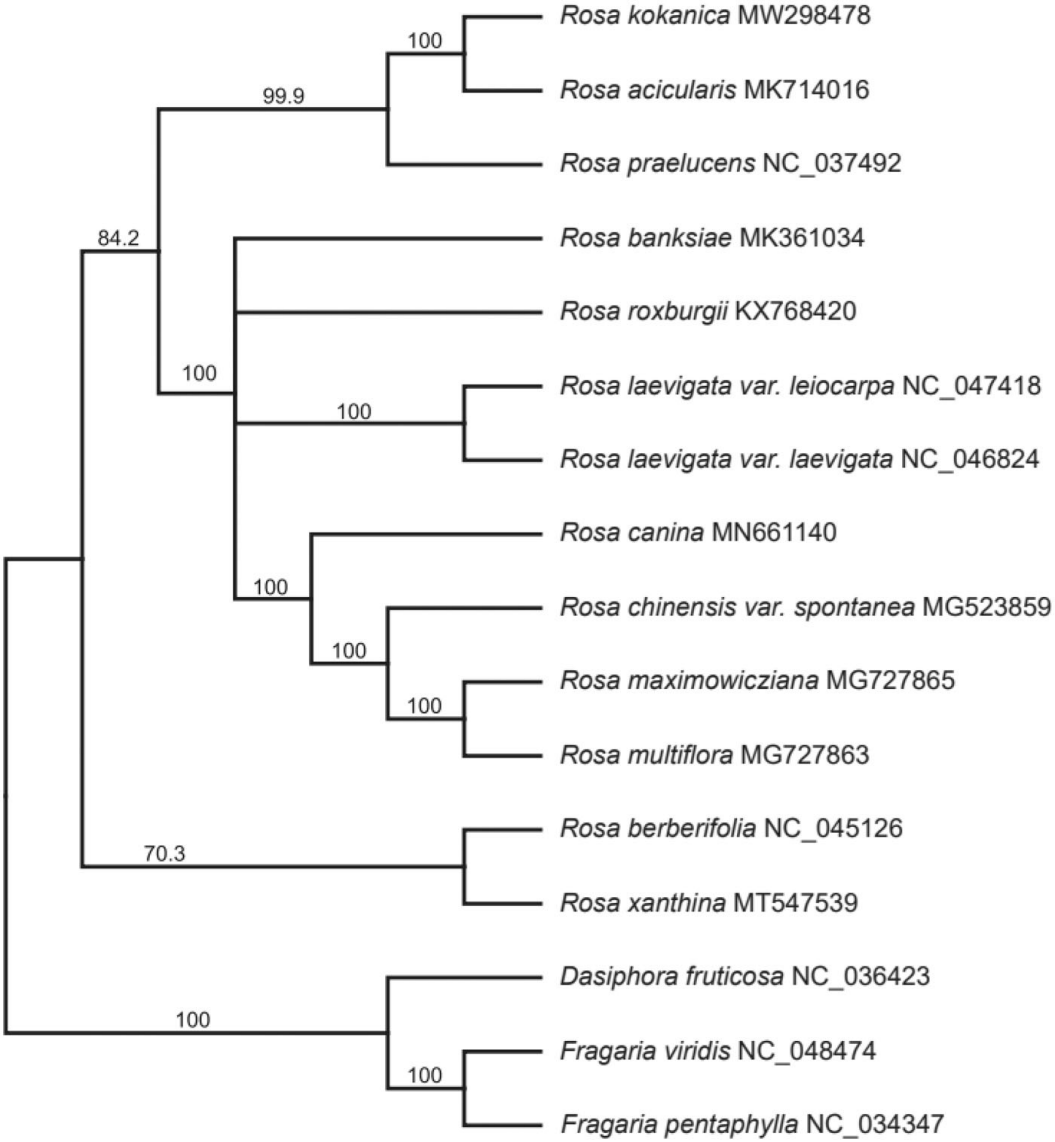
Maximum Likelihood (ML) tree based on chloroplast genome sequences of 16 species of Rosaceae, numbers on the nodes indicated the bootstrap support value (>50%).

## Data Availability

The genome sequence data that support the findings of this study are openly available in GenBank of NCBI at (https://www.ncbi.nlm.nih.gov/) under the accession no. MW298478. The associated BioProject and Bio-Sample numbers are PRJNA706063 and SAMN18115976, respectively.
